# Optical diffraction phenomena around the edges of photodetectors: A simplified method for metrological applications

**DOI:** 10.1038/s41598-019-40270-w

**Published:** 2019-03-04

**Authors:** T. Santhanakrishnan, R. Rajesh, Ram Lochan Awasthi, C. V. Sreehari

**Affiliations:** Naval Physical and Oceanographic Laboratory, Thrikkakara P.O., Kochi, 682 021 Kerala India

## Abstract

An optical method for simultaneous generation and detection of diffraction of light around the edges of photodetectors is reported in this paper along with its application for vibration sensing. The method makes use of an innovative illumination of light beam in which the laser light is made to incident at the interface between the active and opaque regions of a photodetector. Diffraction and induced interference occur together at the sensing area of the photodetector. The same photodetector responds to the dynamic intensity variations corresponding to the diffraction induced interference pattern and concurrently generates a dynamic electrical output. Comparing to the established diffraction techniques employing edges, the proposed method is simple to implement and extends the measurement applications. The experimental results obtained here verify the efficacy of the proposed method indicating its suitability for a novel class of sensors to be employed in practical circumstances.

## Introduction

Lasers are predominantly used as diagnostic tools or as energy sources in scientific research exploration. Controlling the profile of a laser beam in space and time is an important research challenge in optical technology. When the laser beam is used for measurement applications, the spatial profile of the laser beam exhibiting particular distribution patterns and propagation properties in space and time are much more important. The most striking examples are the interference and diffraction patterns often seen every day in experiments with light. In reality, there is no difference between the interference and diffraction pattern. The diffraction pattern seen on any observation screen is really another interference pattern. However, the two phenomena are so different and are so adequately explained in many text books. Daniel Malacara^1^ in his book detailed these phenomena, their differences, advantages and disadvantages and various instruments that are made for physical measurements. Measurements of displacement and vibration have been an area of interest in many engineering problems using these two phenomena.

Several techniques that use optical interference for the measurement of displacements and vibrations have been developed^2^. Numerous traditional interferometers^1^ are now in use for the mission of common man from research laboratories to flying satellites. These optical techniques combined with advanced computers, frame grabbers and image processing algorithms make them handy for most of the industrial applications. Among the classic interferometric techniques^1^ like Moiré interferometery^3,4^, holographic interferometry^5,6^, laser doppler vibrometery^7^, speckle interferometry^8,9^ for vibration monitoring, Michelson interferometer^10^ is the most popularly adopted apparatus by scientists and engineers. These interferometric techniques are considered to have high performance and generally well-suited, and reliable for metrological applications. However, severe drawbacks are associated with their practical use, especially when several measurement points are considered or the installation must be performed in open spaces. Indeed, sensor systems that use interferometry are bulky as they are associated with a number of optical elements and the complexity grows substantially and imposes stringent mechanical requirements because the alignment is critical. Therefore, they are very expensive.

Similarly, several techniques that use optical diffraction^11,12^ have been developed as an alternative to optical interferometry. Different notions are used to describe the phenomenon of diffraction as interference^13,14^ but the utilization of it notably to vibration measurement has still not been fully exploited. Several researchers have contributed to the study of edge diffraction phenomena. The developed theories have been applied to many problems of scientific and engineering interest^15–20^. However, the problem of edge diffraction did not receive significant identity in metrological applications like its counterpart of conventional interferometry mainly because of its geometrical constraints. The difficulty in introducing an opaque edge in the light path is the key geometrical constraint from a system perspective. This paper introduces a relatively simple and efficient method in which the need for an external opaque edge is circumvented. The outer edge of the sensing region of photodetector itself is considered to act as the external opaque edge in the proposed technique. Thereby, simultaneous generation and detection of the optical diffraction pattern are realized using a single photodetector. This simplified technique is quite stable optically and may broaden the scope of the applications of optical diffraction to another stature. Effective utilization of the proposed method is demonstrated as an optical microphone, and the obtained results are verified with a standard microphone.

## Methods

### Concept of edge diffraction

**Figure 1:**
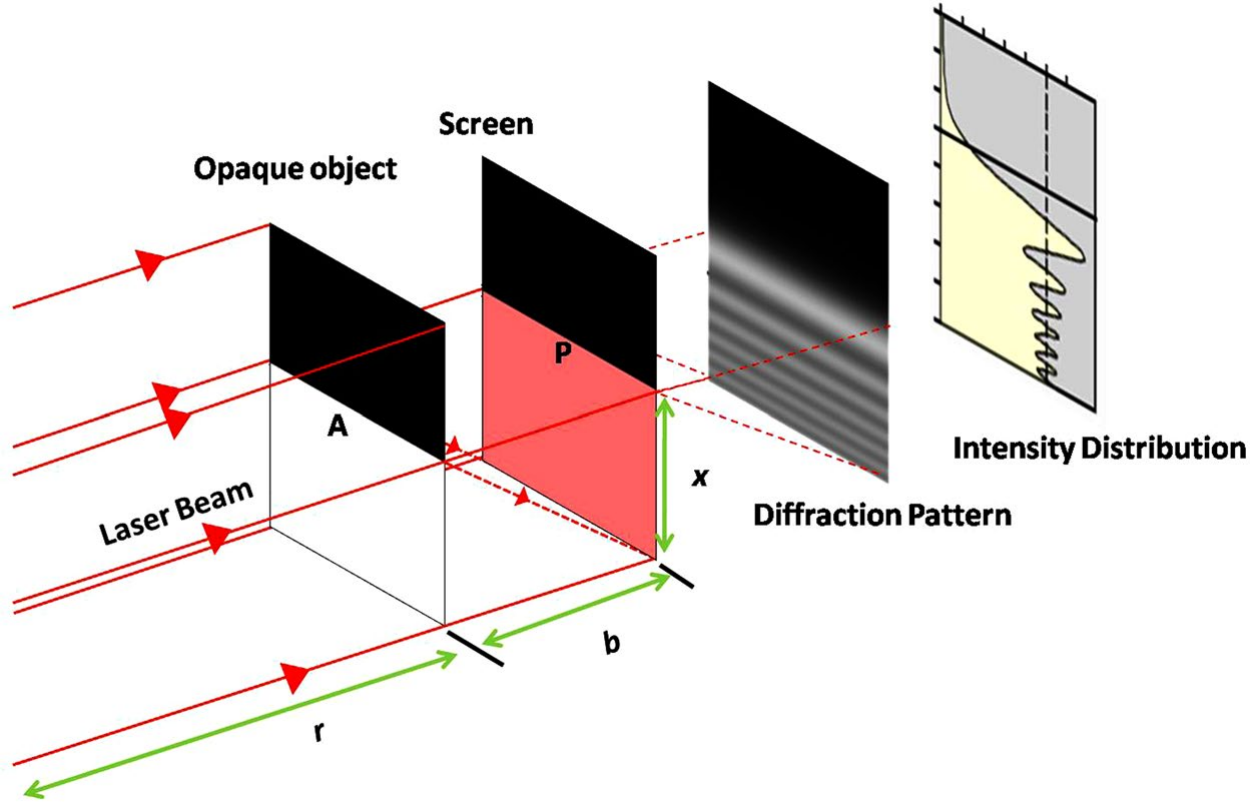
Schematic diagram illustrating the diffraction phenomenon by a straight edge.

Optical diffraction is very often referred to the bending of light waves around an obstacle. Figure 1 schematically illustrates the concept of generating an optical diffraction pattern from a monochromatic light. A light wave is partially blocked by an opaque object before falling onto a screen as shown in Fig. 1. According to the established geometrical theories of optics, the sharp edge of the opaque object casts a shadow having a fairly sharp outline of the same shape as the opaque object at point P. However, by closer inspection, one finds that the edge of this shadow is not absolutely sharp but shows a system of dark and bright bands in the immediate neighbourhood of the edge at the point P. These dark and bright bands are called diffraction pattern as illustrated in Fig. 1. This pattern is due to the diffraction of light around the bottom edge of the opaque object and the result of interference between the direct and the diffracted light rays. If the irradiance at any point is either zero or minimum, it is termed as destructive interference, which represents a dark fringe and if it is maximum it is termed as constructive interference, which represents a bright fringe. Further treatments on this traditional diffraction method are available in the Physics text book by Serway and Jewett^21^. The traditional edge diffraction pattern and intensity distribution shown in Fig. 1 are used later in this paper for comparison of our proposed concept.

The phenomenon of diffraction is studied under two classes, namely Fresnel and Fraunhofer diffraction theories^22–24^. Fresnel diffraction constitutes the poor approximation – cases in which either or both source and observing screen are close enough to the aperture that wave front curvature must be taken into account. The work presented in this paper deals with Fresnel diffraction. Fresnel diffraction pattern is mathematically complex in nature^13,25^.

Among these, Fresnel’s approach based on Huygens’s principle is well known^26,27^. It describes the diffraction field in terms of superposition of two waves: one wave propagates through the diffracting object without any perturbation (called the geometrical wave) and the second wave originates from every point of the illuminated boundary of the object or edge (called the boundary diffracted wave). The intensity distribution at the observation screen can be written as^27^

1 $$I={|{U}^{({\rm{g}})}|}^{2}+{|{U}^{({\rm{d}})}|}^{2}+2{U}^{({\rm{g}})}{U}^{({\rm{d}})}\,\cos \,(k\delta ),$$

where *U*^(g)^ and *U*^(d)^ are the amplitudes of the geometrical and diffracted waves, respectively, *δ* is the phase difference between the geometrical and diffracted waves. Equation (1) reveals that the intensity distribution at the observation plane oscillates between the envelopes ${[{U}^{({\rm{g}})}+{U}^{({\rm{d}})}]}^{2}$ and ${[{U}^{({\rm{g}})}-{U}^{({\rm{d}})}]}^{2}$, it touches the former when cos(*kδ*) = +1 and later when cos(*kδ*) = −1. This shows that in the observation plane, interference fringes are generated in the directly illuminated region where *U*^(g)^ interferes with *U*^(d)^, however, in the geometrical shadow region only *U*^(d)^ is present and thus no interference is observed. The contrast of these fringes goes on decreasing away from the boundary of the shadow. This is because the amplitude of the geometrical wave is almost constant in the directly illuminated region while the amplitude of the boundary diffraction wave falls off rapidly with the distance from the boundary shadow. The fringe width *β* for a bright fringe or a dark fringe for a particular fringe order *n* is^27^

2 $$\beta ={[2\lambda b(r+b)/r]}^{1/2}\,[{(n+1)}^{1/2}-{n}^{1/2}]\,n=1,\,2,\,3,\ldots ,$$

where *r* is distance between the source and the opaque edge and *b* is the distance between the opaque edge and the observation screen. Equation (2) reveals that the fringe width *β* goes on decreasing with increasing fringe order *n*. Further, for a particular fringe order *n*, *β* can be increased by decreasing *r*. Equation (2) also reveals that it can be used to describe the phenomenon based on a point source illumination and not based on a collimated light source. Later in the discussions section, we narrate that the light source used was a diode laser. It has a micro lens attached to its emission head to make the shape of the beam from elliptical to circular and has a divergence of about 0.8 mrad. We have not used any external optics to collimate the laser beam in our experiments. Therefore, Equation (2) can still be used to describe the phenomenon as the laser source with 0.8 mrad divergence can still be approximated to a point source. On the other hand, divergence plays a crucial role in diffraction experiments and hence it cannot be neglected unlike interference experiments. However, the obtainable change in fringe width by changing *r* would be not substantial if the distance between the opaque edge and the observation screen *b* is very less compared to *r*. In the proposed edge diffraction method, *b* is very less compared to *r*.

### Concept of diffraction around a photodetector edge: Simultaneous generation and detection

**Figure 2:**
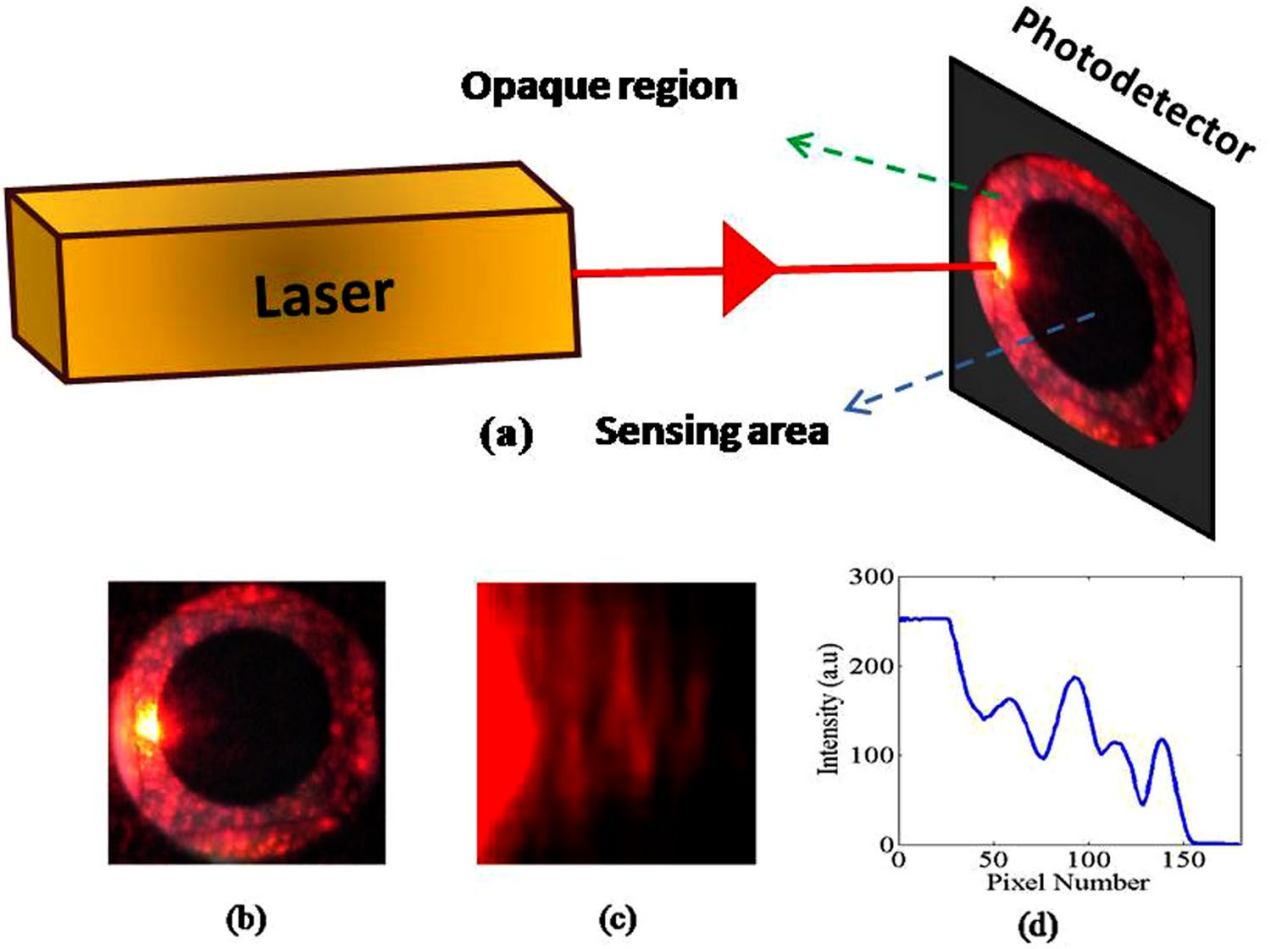
Diffraction around the edge of a photodetector: (**a**) Experimental scheme for simultaneous generation and detection; (**b**) Edge illumination; (**c**) Obtained diffraction pattern and (**d**) Obtained intensity distribution.

In this work, the optical diffraction is established without the support of any external opaque object. On the other hand, the edge of the sensing region of a photodetector, which is used for detecting the diffraction pattern, was used to serve as an opaque object. The experimental setup used to simultaneously generate and detect such a diffraction pattern is shown in Fig. 2(a). Diffraction pattern is generated on a photodetector by illuminating the photodetector such that the light beam incident at the interface between the active sensing and opaque regions of the photodetector as shown in Fig. 2(b). Diffraction pattern formed on the photodetector is shown in Fig. 2(c) and the corresponding intensity distribution obtained along the central horizontal axis is shown in Fig. 2(d). The photodetector concurrently generates an electrical signal corresponding to the fringe pattern in real time. Therefore, the method is said to be simultaneous.

The images presented in Fig. 2(b,c) are recorded by an external Nikon D5600 DSLR camera. The image presented in Fig. 2(b) cannot show the diffraction phenomenon with the details and illumination range directly to an eye but it can indicate the general nature of effect. Image shown in Fig. 2(c) is an enlarged portion of edge diffraction area on photodetector. Subsequently, the image shown in Fig. 2(c) is subjected to analysis using Matlab software. The intensity values are converted to digital information by carefully reading the coordinate points. The data presented in Fig. 2(d) is obtained by retrieving the intensity values along the central horizontal axis of Fig. 2(c).

Obtained fringe pattern and the intensity distribution shown in Fig. 2(c,d) by the proposed method are compared with that of the traditionally established straight edge Fresnel diffraction^21^ shown in Fig. 1. The comparison verifies that there exist a similarity and close agreement between both the traditional and the proposed edge diffraction cases. Fringes obtained by the proposed method are observed to be curved and alternate fringes reveal higher intensity than the previous ones. The reason for this curved appearance of fringes and change in amplitude of alternate fringes is due to the fact that the edge of the photodetector used in this study is not exactly a single straight edge but a curved edge. The curved edges can be treated as comprised of infinite number of infinitesimal straight edges having different orientations. Because of this, different straight edge diffraction patterns are formed in different orientations inside the photodetector and interfere among themselves. This is more common in diffraction problems and is called caustic effect^28,29^. The comparison qualitatively demonstrated that the similarities far exceed the differences except the obvious curvature appearance. No other direct comparison exists in traditional literature and art. However, the studies conducted by P. M. Rinard^30^ in 1976 on large scale diffraction patterns from circular objects also supports the data presented in Fig. 2(d). To further substantiate this data, an equivalent Moiré like pattern can be considered by drawing different straight edge fringes on the observation screen all along the orientation of the curved edge, which will result the data similar to the one shown in Fig. 2(c).

Numerous advantages exist with the proposed edge diffraction system over the other traditional techniques like interferometers. The prime advantage of this method is its simplicity of implementation, where in there is no necessity of optics. Traditional methods involve the use of high-quality optical elements like the lens, beam splitter, mirror and hence there exists associated complications like alignment, aberration and astigmatism kind of optical issues. Traditional interferometers require relatively high power laser sources as the light passes multiple paths through beam splitters and hence associated losses in light intensity are inevitable. Other important advantages are (i) it is cost effective and less sensitive to environmental and thermal issues as no additional optics are involved, (ii) possible to estimate the radius of curvature of the curved diffracting edge, (iii) does not depend on the properties of the diffracting edge material whether it is conducting or non-conducting, reflecting or absorbing and metallic or non-metallic etc. The most spontaneous inherent advantage is that the proposed edge diffraction system has comparable higher diffracted wave intensity with the direct geometrical wave and hence clear fringe patterns can be observed as the diffracting edge is inbuilt within the photodetector observation screen.

The proposed method has additional advantages compared to a quadrant detector system. The quadrant detector has four cells separated by a gap. The basic requirement to use this detector is that the light beam needs to be illuminated in all the four quadrants simultaneously. This condition introduces certain limitations in sensitivity, accuracy, dynamic range and hence the applications.

The method discussed above in this paper finds a number of metrological, civil and defence applications. Some of the most important and striking examples are (i) remote monitoring of acoustic signal, (ii) apparatus to work as an optical microphone, (iii) non-contact apparatus for measuring displacements of objects, (iv) apparatus to work as an optical hydrophone for underwater acoustic detection, (v) apparatus for eavesdropping, (vi) apparatus for detection of surface waves, internal waves and any hydrodynamic disturbances in the ocean, (vii) apparatus for gravitational wave detection (viii) apparatus for detecting pressure vibrations in any optically transparent medium (ix) apparatus for survivor detection from inaccessible rubbles caused by natural calamities etc.

## Results and Discussion

While discussing the proposed edge diffraction system, it is an obvious interest to know about the effect of exposure area between the opaque edge region and the active sensing region. The effect depends on several factors like laser beam size, angle of edge illumination and hence the angle of diffraction and the alignment of the photodetector in addition to the exposure area ratio. The detailed study and the demonstration of this effect involve a large volume of study and hence not attempted in this work.

A realistic 50:50 exposure conditions were followed all through the experiments reported in this paper. However, we made a logical analysis to reasonably address this important effect as it would be beneficial to the readers. The most possible and prominent result due to the influence of exposure area would be an increase and decrease in electrical signal output of the photodetector. The following assumptions are made to explain this effect (i) supposing if the exposed area of the active sensing region is more (i.e. 25:75 exposure conditions), then the obvious expectation is that the first order diffraction fringe will have more intensity and henceforth with the other fringes. However, it is rather difficult to precisely estimate this intensity changes as the exposure area of the diffracted beam inside the photodetector depends on the angle of illumination and the alignment of photodetector. But, it is certain that the net electrical signal output from the photodetector will increase due to the integrated effect of the above explained facts. (ii) The reverse is the case if the exposed area of the active sensing region is less (i.e. 75:25 exposure conditions).

It is also interesting to highlight the effect due to the spatial non-uniformity as most of the photodetectors do suffer issues related to such non-uniformity. The spatial non-uniform responses arise due to several factors such as crystal structure, fabrication quality, radiation, heat conduction and convection losses, inhomogeneities of the material and surface recombination effects, etc. It is also found to be wavelength dependent since absorption strongly depends on the wavelength. Absorption in the visible region becomes moderate enough in silicon photodetectors for photons to reach the depletion region and therefore carrier generation from the depletion region becomes dominant and result in good uniformity^31,32^. We conducted a qualitative analysis on this effect and found that the non-uniform spatial responsivity in silicon photodetectors is less in the visible region with moderately low input laser intensity. However, for higher laser intensity, the non-uniform responses arise even in the visible region. We have verified this effect on the same photodetector with a laser input intensity of 4.9 mW and observed that there are no considerable changes in response over its active sensing surface region. Hence, the spatial non-uniformity effect would not contribute any significant error in our measurements^31,32^.

The experimental method and fabricated sensor were preliminarily characterized in the laboratory and then compared with traditional method and sensors. In the following section, experimental results are reported and discussed.

### Laboratory characterization for vibration sensing

**Figure 3:**
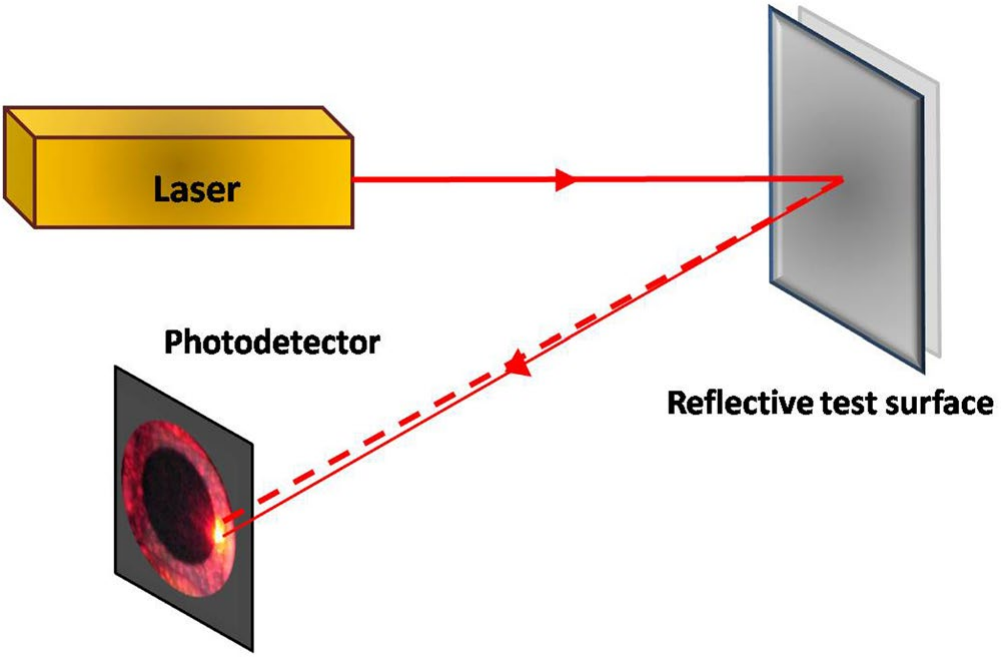
Experimental setup for vibration sensing.

To demonstrate the capability of the method to fitting applications, an appropriate vibration sensing example is chosen for demonstration in this paper. The functionality of the vibration sensing using photodetector edge diffraction is realized on an optical table in the laboratory. Specifically, the dynamic response was tested and compared with that of the traditional method. The experimental setup shown in Fig. 3 is used for this purpose. Here, the laser beam is illuminated on a reflective vibrating test surface and the reflected light beam is collected on the photodetector edge as described in previous paragraphs. In this set-up, a tiny reflecting mirror, which is mounted on the central diaphragm of a laboratory loudspeaker, is chosen as the vibrating test surface. A circular beam diode laser having power of 4.9 mW and wavelength of 635 nm, which belongs to the VHK^™^ class manufactured by M/s. Coherent Inc., USA, was used as the light source. A silicon photodetector having 100 mm^2^ circular sensing area with BNC connector output obtained from M/s. Edmond Optics was used in the experiment. Any inherent or induced vibrations manifested on the diaphragm changes the path of the laser beam which in turn manifests as a time varying diffraction pattern on the photodetector. In this case, the mere path length change will not change the diffraction pattern. The same photodetector detects the temporal changes in the light intensity due to the time varying diffraction pattern, which in turn is the manifestation of the vibrations in real time.

**Figure 4:**
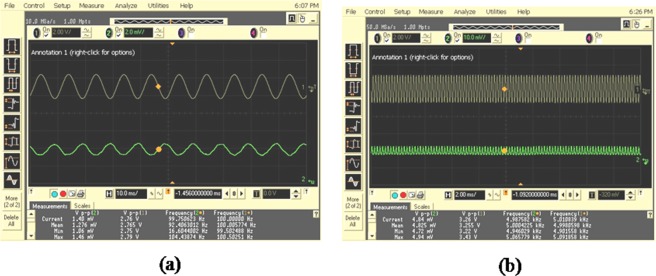
Experimental results validating the capability of the proposed method for vibration sensing and comparison between the reference and observation signals. (**a**) 100 Hz signal and (**b**) 5 kHz signal input to the loud speaker.

A known reference test signal using signal generator is given to the loudspeaker, making the diaphragm of the loudspeaker to vibrate in response to the reference signal. The intensity of the diffraction pattern generated on the photodetector gets modulated at the same rate (amplitude and frequency) of vibration. The photodetector output is connected to one of the channels of an oscilloscope and the reference test signal is connected to another channel for validating purposes. Specifically, the experimental characterisation is extended up to a frequency of 20 kHz, which is a spectral range of utmost importance for acoustic vibration sensing. Two typical results of this characterisation experiment in the form of screen shots are shown in Fig. 4.

In Fig. 4, yellow colour plot corresponds to the signal generator input and green colour plot corresponds to the output of the proposed method. It is observed that the frequencies measured by the oscilloscope for the reference signal and photodetector output by the proposed method are in close agreement and consistent.

### Validation of vibration sensing with multiple frequencies

**Figure 5:**
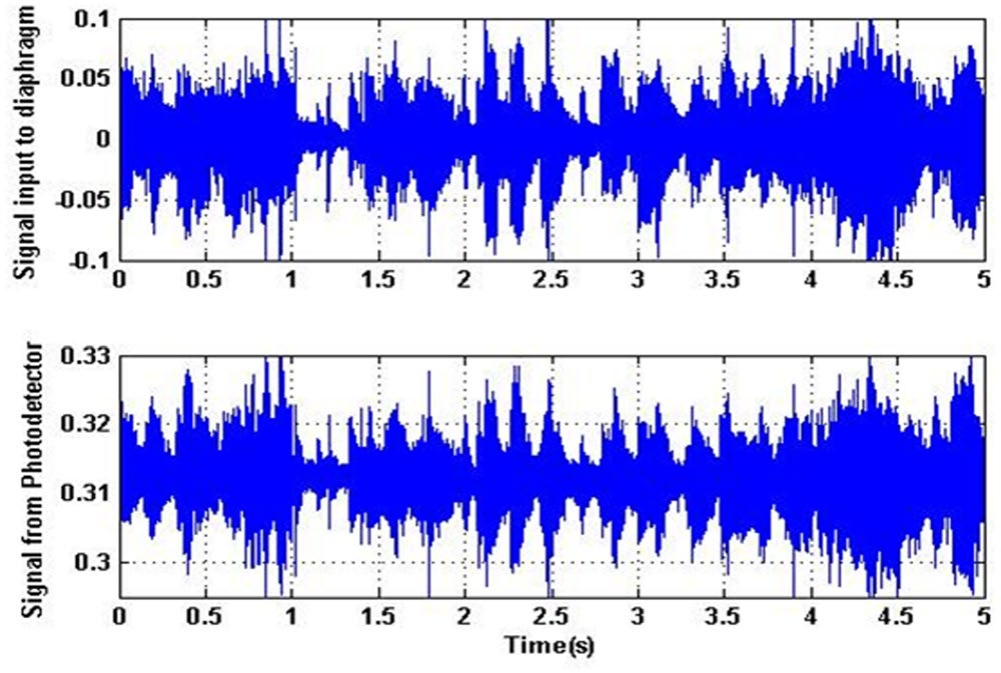
Experimental validation of the proposed method as a vibration sensor for a band of frequencies with an audio wave file input.

To further verify the concept for monitoring vibration with a spectrum of frequencies in real time, an audio wave file is played on a computer and the output is connected to the loud speaker. The experimental setup shown in Fig. 3 was used for this validation experiment. The photodetector output is connected to one of the channels of NI PCI 6014 data acquisition (DAQ) card from National Instruments, which is installed on another PC and the audio wave signal is also given to another channel of the NI PCI 6014 DAQ card. Instantaneously, the data inputs given to the NI PCI 6014 DAQ card are acquired and stored using LabVIEW software. The obtained time series results are shown in Fig. 5.

It is learnt from Fig. 5 that the output signal recorded by the proposed method is in good agreement and consistent with the given signal. This validates the efficacy of the proposed method for sound and vibration monitoring and its capability of detecting and distinguishing multiple frequencies simultaneously.

### Validation as an optical microphone

**Figure 6:**
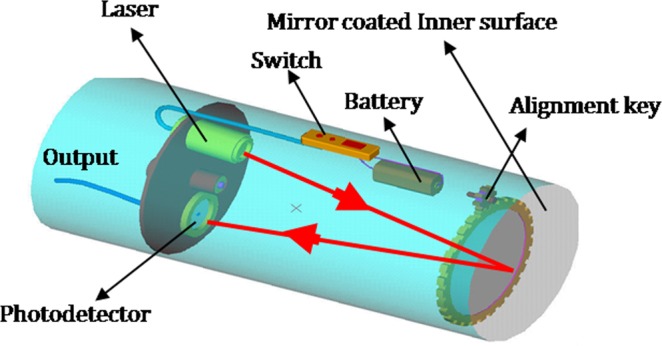
Conceptual design sketch of an optical microphone that uses photodetector edge diffraction.

To demonstrate the functionality of the proposed method as an optical microphone, a compact prototype instrument was designed and fabricated exploiting the method shown in Fig. 3. The conceptual design sketch used for this instrument is shown in Fig. 6. The laser and the photodetector are mounted inside a cylindrical housing with associated battery supply and switch. The front face of the cylindrical housing was fixed with acoustically sensitive material with the inner face reflective coated and an external rotating key mechanism for alignment purposes.

**Figure 7:**
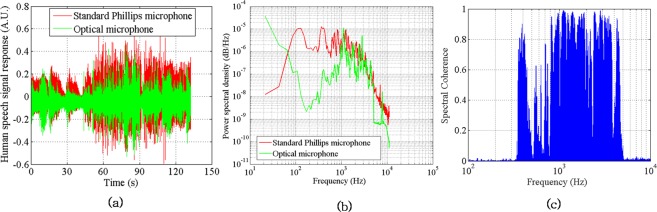
Temporal, spectral and coherence responses of the optical microphone. (**a**) Time response; (**b**) Spectral response and (**c**) Spectral coherence.

The dynamic response of the sensor was verified with a human speech signal. The developed optical microphone sensor and a standard microphone (Manufactured by M/s Phillips, Model: SBCMD150/01) were positioned in front of a person at a distance of 15 cm and his speech signal was recorded and retrieved with the support of an NI PCI 6104 DAQ card and LabVIEW software. Subsequently, this time series data was subjected to spectral analysis using PWELCH and BICOHER functions in Matlab to have an understanding of the spectral response and coherence properties between the developed optical microphone sensor and the standard microphone. Figure 7 shows the typical time series, spectra and spectral coherence responses of the optical and standard microphones. It is observed from the plots that the optical microphone exhibits a comparable frequency response except small deviations in the amplitude. The amplitude features differ because of the inbuilt preamplifier present in the standard microphone. Coherence is found to be consistent in the frequency range 600–4000 Hz demonstrating the fidelity of the method to function as an optical microphone. The other oscillations found in the spectral plot can be attributed to the slightest oscillations emanating from the extraneous sources in the laboratory.

It is worth to note that there exist specific applications, where permanent recordings of signals detected by the instrument such as the one shown in Fig. 6 are required. This can be achieved by connecting the photodetector output terminals shown in Fig. 6 to any other standard audio recorder so that it can be played back to reproduce the recorded signal. On the other hand, the photodetector output terminals can be connected to a standard speaker system with a reasonable amplifier and the input signal given to the optical microphone can be heard in live clearly.

## Conclusions

A novel observation of optical diffraction phenomena around the photodetector edges has been successfully demonstrated. Diffraction fringes are generated and concurrently detected by the same photodetector by illuminating the light beam at the interface between the sensing and opaque regions of the photodetector. This simplifies the traditional edge diffraction problems and thereby expands the utility to sound and vibration sensing applications. The obtained diffraction fringes and the intensity distribution were verified with that of the traditional methods and found to be consistent. To demonstrate the capability of the method for vibration sensing, the sensing system was characterised in the laboratory for a range of frequencies from 100 Hz to 10 kHz. The utility of the method has been exploited to different vibration sensing applications and an optical microphone sensor was fabricated and demonstrated successfully. It is envisaged that the concept and methodology proposed in the paper can find potential civil and defence metrological applications especially in underwater sensing in the near future.

## Data Availability

The authors confirm that the data supporting the findings of this study are available within the article. However, any datasets generated and/or analysed during the current study are available from the corresponding author on reasonable requests.

## References

[1] Malacara, D. *Optical Shop Testing*. 3rd ed. (Wiley-Interscience, John Wiley & Sons, New Jersey, 2007).

[2] Malacara D (1989). Interference: Physical Optics and Light Measurements. Methods in Experimental Physics.

[3] Harding KG, Harris JS (1983). Projection Moiré interferometer for vibration analysis. Applied Optics.

[4] Forno C (1988). Deformation measurement using high resolution Moiré photography. Optics and Lasers in Engineering.

[5] Vest, C. M. *Holographic interferometry*. (John Wiley and Sons, New York, 1979).

[6] Kumar UP, Kalyani Y, Krishna Mohan N, Kothiyal MP (2009). Time-average TV holography for vibration fringe analysis. Applied Optics.

[7] Halliwell NA (1979). Laser-doppler measurement of vibrating surfaces: A portable instrument. Journal of Sound and Vibration.

[8] Høgmoen K, Løkberg OJ (1977). Detection and measurement of small vibrations using electronic speckle pattern interferometry. Applied Optics.

[9] Santhanakrishnan T, Palanisamy PK, Sirohi RS (1998). Fringe formation in an in-plane displacement measurement configuration with twofold increase in sensitivity: theory and experiment. Applied Optics.

[10] Pisani, M. A homodyne Michelson interferometer with sub-picometer resolution. Measurement Science and Technology **20**, 084008 (6pp) (2009).

[11] Malacara D (1989). Diffraction and Scattering: Physical Optics and Light Measurements. Methods in Experimental Physics.

[12] Gåsvik, K. J. *Optical Metrology*. (John Wiley & Sons, New York, 1987).

[13] Born, M. & Wolf, E. *Principles of optics*. 7th ed. (Cam University Press, Edinburgh, 1999).

[14] Wang S (1995). On principles of diffraction. Optik.

[15] Otis G, Lachambre J-L, Lit WY, Lavigne P (1977). Diffracted waves in the shadow boundary region. Journal of the Optical Society of America.

[16] Ganci S (1989). An experiment on the physical reality of edge-diffracted waves. American Journal of Physics.

[17] Khizhnyak AI, Anokhov SP, Lymarenko RA, Soskin MS, Vasnetsov MV (2002). Structure of an edge-dislocation wave originating in plane-wave diffraction by a half-plane. Journal of Optical Society of America A.

[18] Lit JWY, Tremblay R (1969). Boundary-diffraction-wave theory of cascaded-apertures diffraction. Journal of Optical Society of America.

[19] Langlois P, Lessard RA (1986). Simultaneous laser beam profiling and scaling using diffraction edge wave (DEW). SPIE Proceedings.

[20] Polyanskii PV, Polyanskaya GV (1997). Young hologram – a fifth type of hologram. Journal of Optical Technology.

[21] Serway, R. A. & Jewett, J. W. *Physics for Scientists and Engineers*. 6th Ed. (Thomson Brooks/Cole, Boston, 2004).

[22] Sirohi, R. S. *A Course of Experiments with He–Ne Laser*. (Wiley Eastern Limited, New Delhi, 1986).

[23] Eric Udd. *Fibre Optic Sensors: An Introduction for Engineers and Scientists*. (John Wiley and Sons Inc., New York, 1991).

[24] Pedrotti, F. L. & Pedrotti, L. S. *Introduction to Optics, Second edition*. (Prentice Hall, New Jersey, 1993).

[25] Jenkins, F. A. & White, H. E. *Fundamentals of Optics, Fourth edition*. (McGraw-Hill, New York, 2001).

[26] Jennings JK, McGruder CH (1999). Comparison of the disk diffraction pattern with the straight-edge diffraction pattern in occultations. The Astronomical Journal.

[27] Kumar R, Kaura SK, Sharma AK, Chhachhia DP, Aggarwal AK (2007). Knife-edge diffraction pattern as an interference phenomenon: An experimental reality. Optics and Laser Technology.

[28] Berry MV (1987). Disruption of images: the caustic-touching theorem. Journal of Optical Society of America A.

[29] Berry MV, Wilson AN (1994). Black-and-white fringes and the colors of caustics. Applied Optics.

[30] Rinard PM (1976). Large-scale diffraction patterns from circular objects. American Journal of Physics.

[31] Fischer J, Fu L (1993). Photodiode nonlinearity measurement with an intensity stabilized laser as a radiation source. Applied Optics.

[32] Kübarsepp T, Haapalinna A, Kӓrhӓ P, Ikonen E (1998). Nonlinearity measurements of silicon photodetectors. Applied Optics.

